# Exosomes as drug delivery systems in glioma immunotherapy

**DOI:** 10.1186/s12951-024-02611-4

**Published:** 2024-06-18

**Authors:** Xinqing Hao, Shiming Wang, Liang Wang, Jiaqi Li, Ying Li, Jing Liu

**Affiliations:** 1 Stem Cell Clinical Research Center, The First Affiliated Hospital of Dalian Medical University, No. 193 Lianhe Road, Dalian, Liaoning 116011 China; 2 Dalian Innovation Institute of Stem Cell and Precision Medicine, No. 57 Xinda Road, Dalian, Liaoning 116085 China; 3 Department of Urology, The First Affiliated Hospital of Dalian Medical University, No. 193 Lianhe Road, Dalian, Liaoning 116011 China; 4 Reproductive Medicine Center, The First Affiliated Hospital of Dalian Medical University, No. 222 Zhongshan Road, Dalian, 116011 China

**Keywords:** Exosomes, Glioma, Drug delivery systems, Immunotherapy

## Abstract

Recently, the significant benefits of cancer immunotherapy for most cancers have been demonstrated in clinical and preclinical studies. However, the efficacy of these immunotherapies for gliomas is limited, owing to restricted drug delivery and insufficient immune activation. As drug carriers, exosomes offer the advantages of low toxicity, good biocompatibility, and intrinsic cell targeting, which could enhance glioma immunotherapy efficacy. However, a review of exosome-based drug delivery systems for glioma immunotherapy has not been presented. This review introduces the current problems in glioma immunotherapy and the role of exosomes in addressing these issues. Meanwhile, preparation and application strategies of exosome-based drug delivery systems for glioma immunotherapy are discussed, especially for enhancing immunogenicity and reversing the immunosuppressive tumor microenvironment. Finally, we briefly describe the challenges of exosome-based drug delivery systems in clinical translation. We anticipate that this review will guide the use of exosomes as drug carriers for glioma immunotherapy.

**Graphical Abstract:**

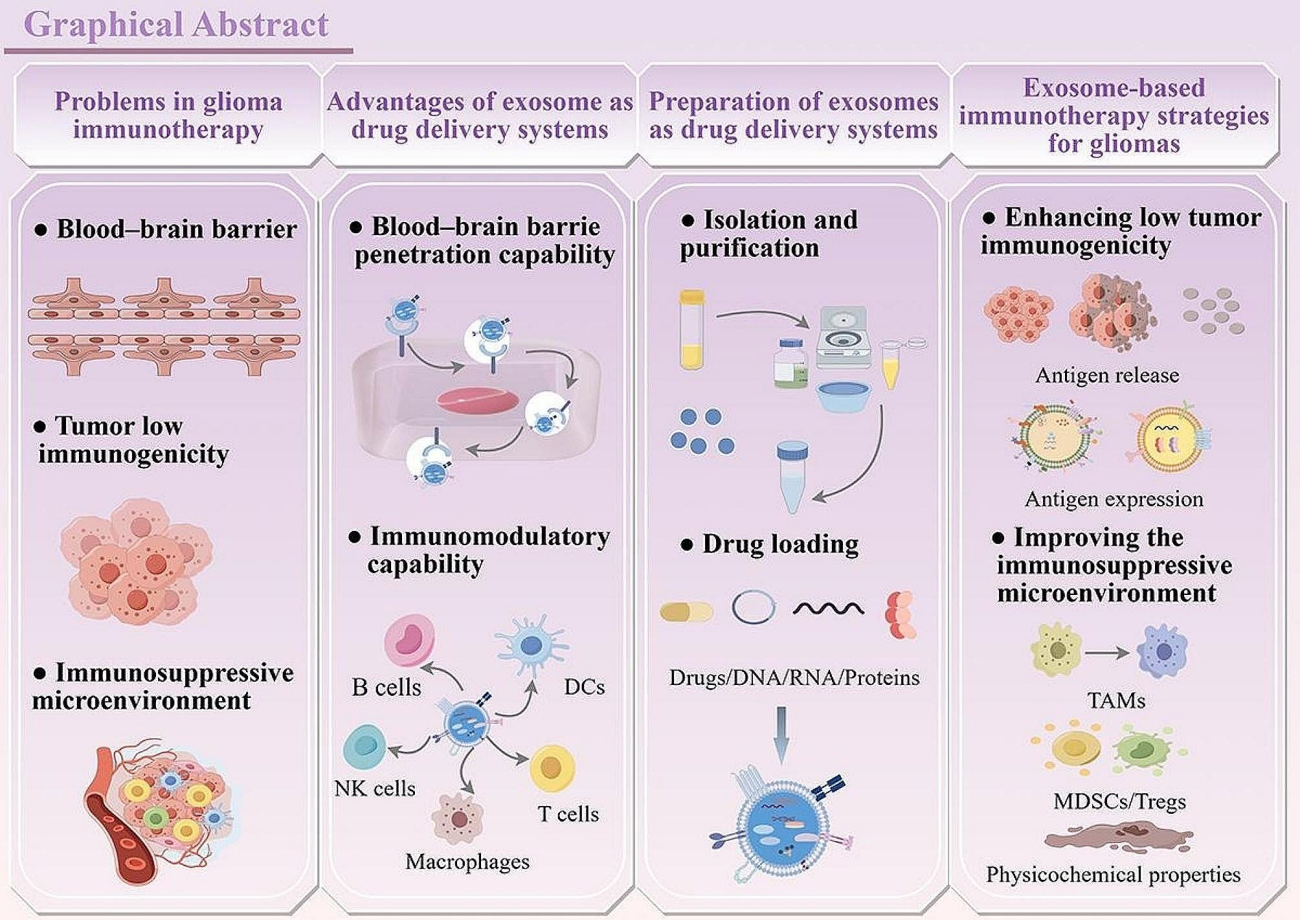

## Introduction

Gliomas are tumors derived from glial or neural precursor cells in the central nervous system (CNS), including astrocytoma, oligodendroglioma, ventricular meningioma, and mixed glioma. According to recent data from The Central Brain Tumor Registry of the United States, gliomas account for 24% of all CNS tumors and 80.9% of malignant tumors, making them the most prevalent intracranial malignancy [1]. The World Health Organization categorizes gliomas into four grades (grades 1–4), with grades 1–2 considered low-grade gliomas and grades 3–4 classified as high-grade gliomas [2]. Low-grade gliomas are associated with a 10-year survival rate of 39% and a median survival time of 87.3 months [3]. Conversely, high-grade gliomas, particularly glioblastoma (GBM), demonstrate high malignancy, with a median survival time not exceeding 15 months [4]. Owing to their high recurrence rate, high disability and mortality rates, and limited cure rate, gliomas are the most aggressive and refractory CNS tumors. Currently, the standard treatment for gliomas involves surgical intervention combined with chemoradiotherapy; however, this approach is inefficient in preventing tumor recurrence. Therefore, it is critical to explore innovative treatment approaches to enhance therapeutic efficacy.

In recent years, tumor immunotherapy has gained prominence for its ability to restore and enhance immune responses, through exogenous intervention with the body’s immune system. The tumor immune cycle includes the release of specific antigens by tumor cells, which are captured and processed by antigen-presenting cells (APCs) for presentation to T cells. These T cells are then activated, and they proliferate and infiltrate tumors, ultimately recognizing and eliminating tumor cells [5]. Tumor immunotherapy can specifically kill tumor cells and establish long-term immune memory by facilitating any step in this process. Common cancer immunotherapies include immune checkpoint inhibitors (ICIs), chimeric antigen receptor T cell therapy, cancer vaccines, and oncolytic viruses, and these are currently the subject of many clinical trials [6]. However, patients with glioma derive limited benefits from immunotherapy compared to those with other malignancies [7]. Several factors contribute to this, including (i) the blood–brain barrier (BBB) preventing the entry of therapeutic molecules, (ii) the low immunogenicity of gliomas limiting the initiation of immune responses, and (iii) the immunosuppressive microenvironment inhibiting the activity of effector T cells. To address these challenges, researchers are developing drug delivery systems capable of efficiently delivering therapeutic agents, such as antigens, immunomodulatory molecules, and anticancer drugs. These systems aim to penetrate the BBB precisely and enhance the immune response, thereby improving the efficacy of glioma immunotherapy.

Common drug delivery systems mainly comprise nanoformulations, including liposomes, solid lipid nanoparticles, dendritic polymers, and inorganic materials [8, 9]. However, these nanoformulations are identified as “non-self” and are susceptible to capture and rapid clearance by the immune system. Additionally, nanocarriers synthesized from inorganic materials can have potential toxicity owing to their inability to be degraded [10]. In contrast, exosomes are endogenous nanoscale vesicles secreted by living cells, with several advantages, including excellent stability, low toxicity, a long circulating half-life, ease of modification, and intrinsic cell targeting. Most notably, exosomes are vital in mediating cellular communication within the tumor immune response. In addition, they participate in presenting immune signals, regulating the tumor microenvironment (TME), and influencing immune cell responses [11]. Therefore, exosomes have great potential as carriers for drug delivery in glioma immunotherapy. However, a review on this topic has not been presented. In this review, we introduce the problems in glioma immunotherapy and the advantages of exosomes in it, and we discuss the preparation of exosomes as drug delivery systems and the strategies for their application in glioma immunotherapy to provide new ideas for realizing efficient glioma treatment.

## Current problems in glioma immunotherapy

### Blood–brain barrier

**Fig. 1:**
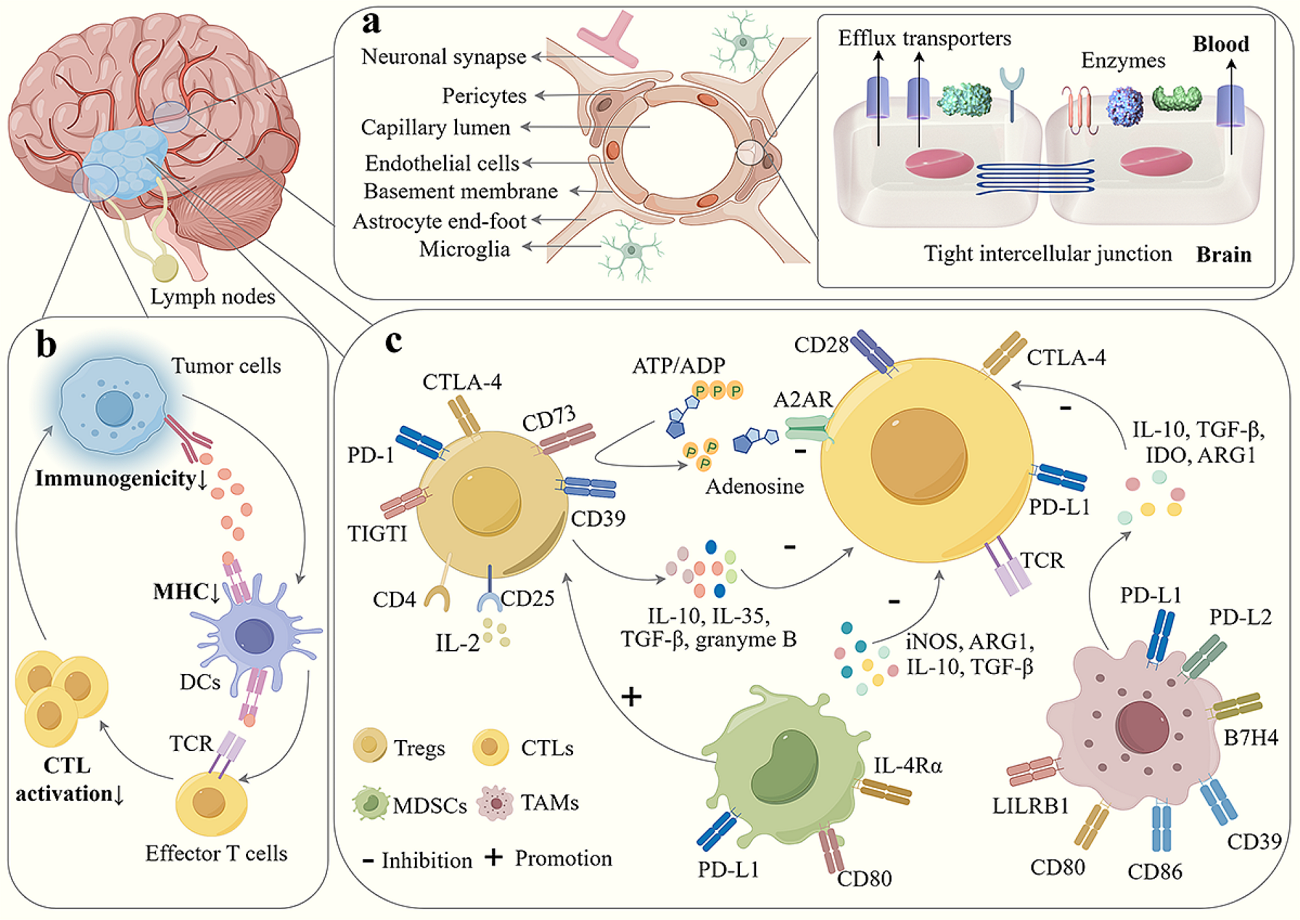
Current problems in glioma immunotherapy (By Figdraw). **a** Blood-brain barrier prevents the entry of therapeutic molecules. **b** Low immunogenicity of gliomas restricts immune response initiation. **c** Immunosuppressive microenvironment inhibits T cell activity

The BBB is a neurovascular unit consisting of brain microvascular endothelial cells (BMECs), basement membrane, astrocytes, microglia, and pericytes [12]. Among these components, BMECs, which form the primary structure of the BBB, display a flattened cell morphology, limited caveolae on the luminal surface, abundant mitochondria, and tight intercellular junctions [13]. This unique physical barrier permits only lipophilic molecules with molecular weights less than 400 Dalton and fewer than eight hydrogen bonds to traverse the BBB along the concentration gradient [14]. In addition, highly active intracellular and extracellular enzymes on the BBB prevent drugs, such as proteins and peptides, from passing through the BBB by degrading them [15]. Furthermore, various transmembrane transporter proteins, particularly ATP-binding cassette transporters, limit the accumulation of certain small-molecule drugs by expelling them into the capillary lumen [16, 17]. Given these constraints, the BBB not only protects the brain from foreign pathogens but also blocks nearly all large-molecule drugs and approximately 98% of small-molecule drugs from reaching the treatment site, while simultaneously controlling peripheral immune cell infiltration into the brain [18, 19] (Fig. 1a). As tumors progress, BBB disruption can lead to the formation of a blood–brain tumor barrier characterized by a looser vascular system, diminished tight junctions, an abnormal pericyte distribution, an altered astrocyte end-foot, and reduced neuronal connectivity [20]. In a sense, BBB disruption might facilitate the entry of drugs and immune factors into tumors. However, the blood–brain tumor barrier still expresses efflux transporters [20], and clinical data indicate that in all patients with GBM, an intact BBB is retained in the tumor region [21]. Thus, drugs that effectively penetrate the BBB are essential for glioma treatment.

### Tumor low immunogenicity

The tumor mutational burden (TMB) indirectly reflects the ability of tumors to produce neoantigens and can be used to predict the therapeutic efficacy of ICIs. A higher TMB suggests that the patient is more likely to benefit from ICIs [22]. However, GBM exhibits a low TMB and neoantigen burden, resulting in limited therapeutic targets for the immune system [23, 24] (Fig. 1b). By evaluating changes in the neoantigen load and immunologic characteristics in patients with primary and recurrent gliomas, Neio et al. [25] found no difference in the number of expressed neoantigens (e-) and predicted neoantigens (p-); however, their ratio (e-/p-) was significantly decreased in patients with recurrent gliomas. They concluded that the reduced expression of mutant neoantigens and impaired antigen processing and presentation could cause tumor immune escape and recurrence. Furthermore, beyond mutated neoantigens, non-mutated shared antigens, such as IL13Rα2, HER2, WT1, and EphA2, are commonly found in most patients with glioma. However, these antigens are expressed at low and highly variable levels compared to those in malignant tumors such as melanoma [26, 27]. At the same time, these non-mutated antigens are also usually expressed in normal tissues [28], increasing the risk of complications when targeting them for treatment.

Additionally, tumor antigens necessitate the formation of mature peptide–major histocompatibility complexes (MHCs) through antigen processing and presentation machinery (APM) for T cell recognition. Previous research has shown that glioma cell migration and invasion are correlated with downregulated MHC class I and II antigen expression [29]. In developing APVAC2 for the GAPVAC-101 clinical trial, based on a vaccine derived from glioma-associated mutant peptides, researchers performed extensive immunopeptidome analysis. They identified 35,156 distinct human leukocyte antigens (HLAs) but found no mutant peptides presented by HLA in the plasma or tissue from patients with GBM [30, 31]. Moreover, Facoetti et al. observed that more than 50% of patients with GBM were deficient in HLA class I and II molecules, with a significant correlation between the loss of HLA class I and a higher tumor grade. Furthermore, the expression of tapasin, a key component of the APM that facilitates MHC class I interactions with antigen-processing transporters, was determined to be reduced in several GBM specimens. This indicates that abnormalities in HLA and APM might contribute to the limited efficacy of immunotherapy for malignant gliomas [32].

### Immunosuppressive microenvironment

Tumor cell invasion into normal tissues establishes the TME, which comprises immune cells, cancer-associated fibroblasts, endothelial cells, and an extracellular matrix [33, 34]. In the TME, densely infiltrating immunosuppressive cells and cytokines, along with hypoxia and chronic inflammation, synergistically inhibit T cell responses, facilitate tumor immune escape, and adversely affect glioma immunotherapy [35, 36] (Fig. 1c). Tumor-associated macrophages (TAMs) constitute 40% of the total tumor volume and play a crucial role in the TME [37]. Accordingly, high TAM infiltration is typically linked to reduced overall survival in patients with GBM [38]. TAMs secrete TGF-β, targeting cytotoxic T lymphocytes (CTLs) and specifically suppressing the expression of perforin, granzyme A, granzyme B, Fas ligand, and IFN-γ, consequently diminishing CTL-mediated cytotoxicity [39]. Moreover, GBM-induced kynurenine activates the aryl hydrocarbon receptor in TAMs, promoting CD39 expression, which synergizes with CD73 to produce adenosine, leading to CD8^+^ T cell dysfunction [40]. Furthermore, TAMs disrupt T cell metabolism through the overexpression of arginase (ARG1) and indoleamine 2,3-dioxygenase (IDO), catabolizing amino acids that are vital for T cell proliferation [41]. Similarly to that observed in tumor cells, TAMs express inhibitory surface molecules, such as PD-L1, PD-L2, B7H4, and CD80/CD86, which hinder T cell activation and even trigger apoptosis [42]. Moreover, the LILRB1 inhibitory receptor on TAMs binds to MHC I on cancer cells, directly shielding them from immune-mediated phagocytosis [43].

Myeloid-derived suppressor cells (MDSCs) represent a diverse group of immature myeloid cells (IMCs) known for their immunosuppressive capabilities [44]. Typically, IMCs originating from the bone marrow rapidly differentiate into mature macrophages, granulocytes, and dendritic cells (DCs) [45, 46]. In contrast, the maturation of IMCs is hindered, leading them to transform into MDSCs with immunosuppressive functions in tumors [47]. MDSCs can pleiotropically inhibit CTL activity through the expression of IL-4Rα, PD-L1, and CD80 and the upregulation of ARG1 and inducible nitric oxide synthase (iNOS) expression [48]. Research has further shown that systemic lymphopenia following radiotherapy is correlated with an increase in MDSC numbers and that targeting MDSCs is effective for mitigating immunosuppression and lymphopenia in patients with GBM patients [49]. Moreover, MDSCs exert immunosuppressive effects by inhibiting the functions of DCs, natural killer (NK) cells, and macrophages, in addition to inducing the expansion and differentiation of regulatory T cells (Tregs).

Tregs are a subpopulation of CD4^+^ T cells that usually express the transcription factor Foxp3. The tumor tissues and peripheral blood of patients with glioma have a higher proportion of Tregs compared to that in healthy individuals [50], with a higher glioma grade corresponding to more significant Treg enrichment [51]. Tregs can suppress anti-tumor immune responses through a variety of mechanisms [52–54], including (i) the secretion of immunosuppressive cytokines, (ii) the induction of target cell lysis via cell–cell contact, (iii) the expression of inhibitory receptors to suppress APCs, (iv) competition with effector T cells for IL-2 to inhibit their growth, and (v) the promotion of adenosine accumulation and competition for nutrients to disrupt T cell metabolism. Recently, van Hooren et al. [55] reported that CD103^+^ Tregs with upregulated lipid metabolism are accumulated in the TME and significantly inhibit CTL activation after radiotherapy combined with ICIs. The depletion of Tregs results in tertiary lymphoid structure formation, which in turn induces responses in CTLs and improves treatment resistance. Therefore, there is an urgent need to develop therapies targeting immunosuppressive cells to reverse the immunosuppressive microenvironment.

## Overview of exosomes

### Biogenesis and characteristics of exosomes

**Fig. 2:**
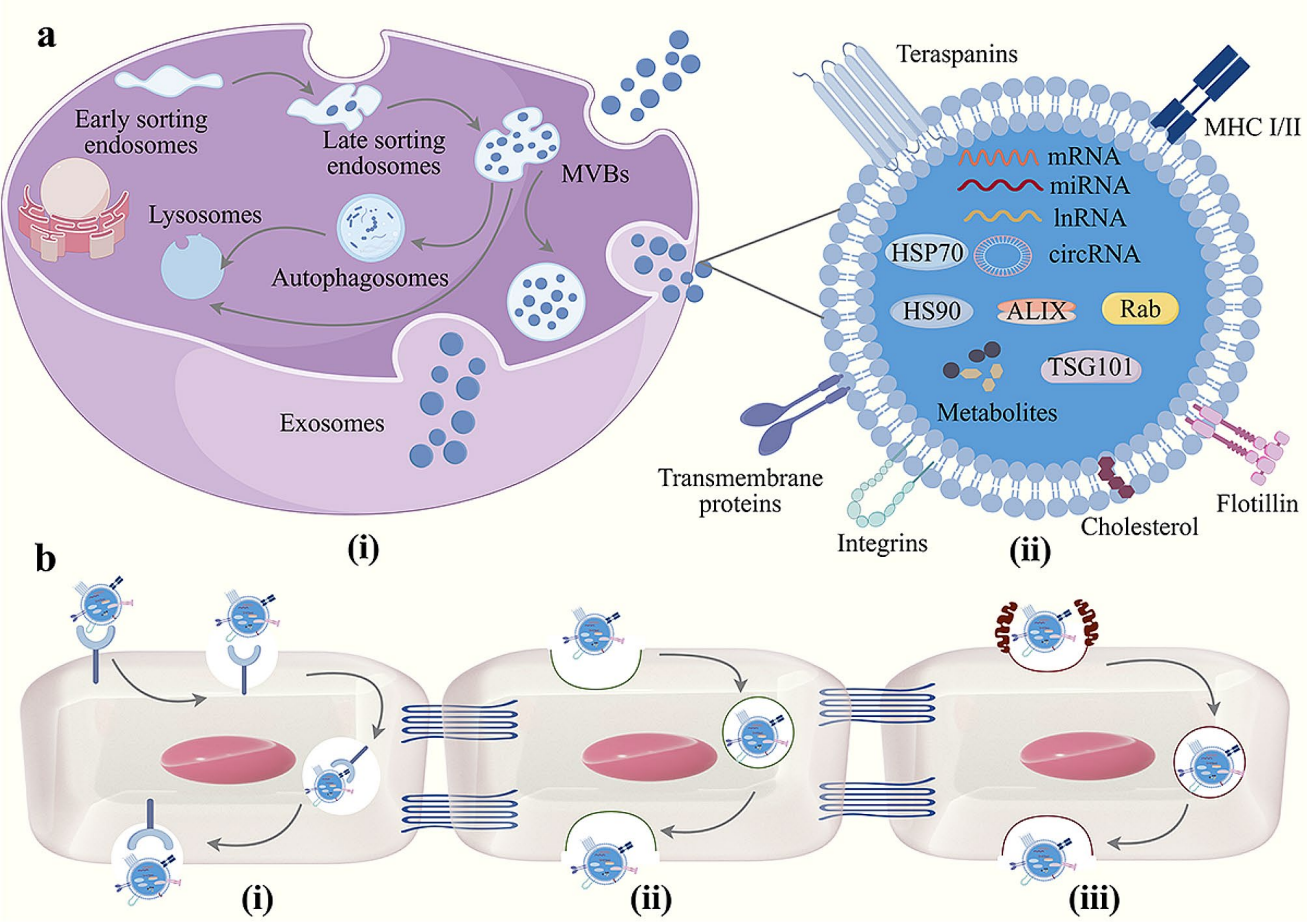
Exosome biogenesis, characteristics, and mechanism of crossing the BBB (By Figdraw). **a** Exosome biogenesis and characteristics. (i) Exosome biogenesis; (ii) Exosome characteristics. **b** Mechanism of exosome crossing the BBB. (i) Receptor-mediated transcytosis; (ii) Lipid rafts or nonspecific exosome-endothelial cell interactions; (iii) Macropinocytosis

Exosomes are double-membrane structured extracellular vesicles with a 40–160 nm diameter formed by outgrowth of the plasma membrane. Most cells can actively secrete exosomes, which are naturally present in various body fluids such as blood, saliva, urine, and cerebrospinal fluid [56]. Exosome generation involves double invagination of the plasma membrane and the formation of intracellular multivesicular bodies (MVBs) containing intraluminal vesicles (ILVs) (Fig. 2a(i)). Specifically, cell membranes undergo endocytosis to form early sorting endosomes, which then mature and further invaginate to form MVBs. Subsequently, MVBs can either be degraded by lysosomes or autophagosomes or fuse with the plasma membrane, thereby releasing ILVs as exosomes [57]. Exosome biogenesis generally involves two potential mechanisms: endosomal sorting complex required for transport (ESCRT) and ESCRT-independent pathway. The ESCRT pathway relies on the synergistic of the ESCRT complexes (ESCRT-0, ESCRT-I, ESCRT-II, and ESCRT-III) and the AAA ATPase Vps4 for recognition sorting of ubiquitinated proteins to maintain the specificity of the contents of ILVs [58]. The ESCRT-independent pathway is driven by partial lipids. Sphingomyelins produce ceramides via neutral sphingomyelinase 2, and ceramides form lipid raft microdomains to trigger the formation of ILVs while promoting exosome release [59].

Depending on the parental cell of origin, exosomes can carry a combination of different bioactive molecules, including proteins, nucleic acids, and lipids (Fig. 2a(ii)) [60]. Among these molecules, proteins constitute the most abundant component of exosomes and carry a wide range of functions. Synaptosomal-associated proteins, annexins, and Rab proteins are responsible for intracellular membrane fusion and trafficking. Heat shock proteins such as Hsp70 and Hsp90 are involved in antigen presentation and immunomodulation, while proteins like Alix and TSG101 are essential for MVB biogenesis. Tetratransmembrane proteins (CD9, CD36, CD81, and CD82) and integrins mediate cell infiltration, adhesion, and fusion. Transporter proteins (ATP7A, ATP7B, SLC16A1, and CL1C1) and receptors (CD46, CD55) facilitate cell communication and molecular transport [61–63]. Another vital cargo of exosomes is nucleic acids, including messenger RNAs (mRNAs), microRNAs (miRNAs), circular RNAs (circRNAs), and long non-coding RNAs (lnRNAs). mRNAs mediate genetic information transfer and regulate receptor cell function and protein synthesis [64]. miRNAs can inhibit the translation of target mRNAs or promote their degradation to regulate gene expression, affecting a wide range of biological processes, such as cell proliferation, differentiation, apoptosis, and disease development [65]. circRNAs and lnRNAs can be selectively packaged, secreted, and transferred between cells in exosomes to regulate cancer angiogenesis, immune escape, invasive metastasis, and epithelial-mesenchymal transition [66, 67]. Furthermore, exosomes contain diverse lipids, such as ceramides, gangliosidesphosphatidylserine, cholesterol, sphingomyelins, phosphatidylcholine, and phosphatidylserine. These lipid components not only keep the morphology and structure of exosomes stable but also play a crucial role in the production and release of exosomes [68, 69].

### Biological function of exosomes

The diversity of the molecular composition of exosomes endows them with a wide range of biological functions that are important for maintaining the internal homeostasis of organisms, influencing the course of diseases, and developing new therapeutic approaches. First, intercellular signaling and communication are critical biological functions of exosomes. Exosomes are taken up by recipient cells through receptor-ligand interactions, membrane fusion, or endocytosis, delivering the bioactive molecules and thus modulating the functional and phenotypic characteristics of the recipient cells [70, 71]. For example, exosomes loaded with the hypoxia master regulator HIF1A transmit hypoxic signals to normoxic cells, thereby inducing neoangiogenesis [72]. Glioma-derived exosomes (GM-Exos) induce the conversion of normal human astrocytes to tumor-associated astrocytes via the miR-3065-5p/DLG2 signaling axis and promote malignant tumor progression [73]. Additionally, exosomes can modulate inflammation by inhibiting or promoting the activation of inflammasomes. Studies have shown that M2-like macrophage exosomes carrying miR-148a inactivate the TLR4/NF-κB/NLRP3 signaling pathway to alleviate myocardial ischemia/reperfusion injury [74], while macrophage-derived exosomes treated with lipopolysaccharides promote acute liver injury by activating the NLRP3 inflammasome [75]. The role of exosomes in immune responses has also been extensively studied. Depending on their molecular composition and the specific cellular environment in which they reside, exosomes can enhance immune responses or induce immune tolerance in their communication with immune cells, demonstrating their potential therapeutic role in cancer, autoimmune diseases, inflammatory diseases, and transplant rejection [76, 77]. Moreover, exosomes exhibit the ability to promote tissue repair and regeneration. In particular, mesenchymal stem cell-derived exosomes can stimulate vascular regeneration, resist apoptosis and fibrosis, enhance neuronal survival, and improve extracellular matrix remodeling [78, 79]. Numerous studies have demonstrated that mesenchymal stem cell-derived exosomes show protective and therapeutic benefits in wound healing, bone and cartilage regeneration, nerve repair, and myocardial injuries [80, 81]. Meanwhile, exosomes can carry specific molecules that reflect the pathophysiological state of their source cells, thus serving as biomarkers for early diagnosis and monitoring of diseases [82]. Finally, based on their molecular transfer function, good biocompatibility, stability, and targeting properties, exosomes have been widely investigated for drug delivery [83].

### Status of exosomes for glioma treatment

In recent years, researchers’ in-depth exploration of exosomes’ composition and function has facilitated their remarkable progress in glioma treatment, mainly divided into four directions. First, inhibiting the release or uptake of GM-Exos to weaken their role in promoting glioma malignant progression. Several potential therapeutic targets have been identified, including ubiquitin-conjugating enzyme E2O, heparan sulfate proteoglycans, and the mammalian target of rapamycin [84, 85]. Second, exosomes’ inherent properties and contents can be utilized to treat gliomas. For example, mesenchymal stem cell-derived exosomes have shown the potential to treat gliomas independently of other therapies [86, 87]. Microglia-derived exosomes stimulated by lipopolysaccharides/INF-γ can also inhibit glioma growth by altering the phenotype of TAMs [88]. Third, exosomes can be used as biomarkers to monitor treatment efficacy and predict prognosis. Studies have demonstrated that certain bioactive molecule levels (e.g., circ_0072083, lncSBF2-AS1, and miR-1238) in the exosomes of glioma patients correlate with the level of temozolomide resistance and treatment efficacy [89–91]. In addition, circulating extracellular vesicle levels can reflect overall survival, progression-free survival, degree of tumor resection, and postoperative recurrence in glioma patients [92]. Finally, and more importantly, exosomes can deliver nucleic acids, drugs, and proteins to treat gliomas, including anti-angiogenesis, inhibiting tumor growth and metastasis, reversing drug resistance, and enhancing immune responses [18]. In the following subsection, we will focus on the advantages of exosomes as carriers in glioma immunotherapy.

## Advantages of exosomes as drug delivery systems in glioma immunotherapy

### BBB-penetration capability

The ability of exosomes to penetrate the BBB and enter the brain has been demonstrated. Banks et al. [93] found that exosomes derived from different species (human and mouse) and cell lines (cancerous and noncancerous) can penetrate the BBB, with penetration rates varying more than 10-fold. Here, lipopolysaccharide and wheatgerm agglutinin were found to regulate the transport of most exosomes, and the mannose 6-phosphate receptor was determined to be a potential receptor facilitating the movement of mouse macrophage exosomes across the BBB. In addition, the investigators outlined possible transport pathways utilized by exosomes to penetrate the BBB, such as macropinocytosis, lipid rafts or nonspecific exosome–endothelial cell interactions, and receptor-mediated transcytosis [94, 95] (Fig. 2b). Although the detailed mechanisms underlying exosome entry into the brain need to be fully elucidated, several studies have indicated that the binding of specific ligands on the surfaces of exosomes to brain endothelial cell receptors facilitates their passage across the BBB. For example, both in vitro and in vivo models have been used to confirm that naïve macrophage-derived exosomes are internalized by BMECs through interactions between surface integrin lymphocyte function-associated antigen 1 and intercellular adhesion molecule 1 (ICAM-1) [96]. Moreover, neural stem cell-derived exosomes that bind heparan sulfate proteoglycan receptors are endocytosed by BMECs and cross the BBB [97]. Further, serum exosomes exhibit natural brain-targeting abilities through transferrin–transferrin receptor interactions [98]. CD46 is one of the primary receptors utilized by BMECs to take up exosomes derived from brain metastatic cancer cell lines and promote cancer metastasis [99]. In addition, different pathologic conditions enhance the uptake of exosomes by the BBB. Inflammation causes the overexpression of ICAM-1 receptors on the BBB, and macrophage exosomes enter the inflamed brain 3.1-fold faster and accumulate 5.8-fold more than in a healthy brain [96]. Hypoxia triggers intracellular calcium release, modifies connexins to disrupt the BBB, and leads to the significant leakage of exosomes into the brain [100]. The brain-targeting capabilities of both natural and engineered exosomes have led to their excellent therapeutic efficacy in models of CNS diseases, including Alzheimer’s disease, Parkinson’s disease, stroke, and epilepsy [94]. Therefore, exosomes offer broad clinical applications for the delivery of drugs to treat CNS diseases and hold promise for overcoming the challenges to glioma immunotherapy posed by the BBB.

### Immunomodulatory capability

**Fig. 3:**
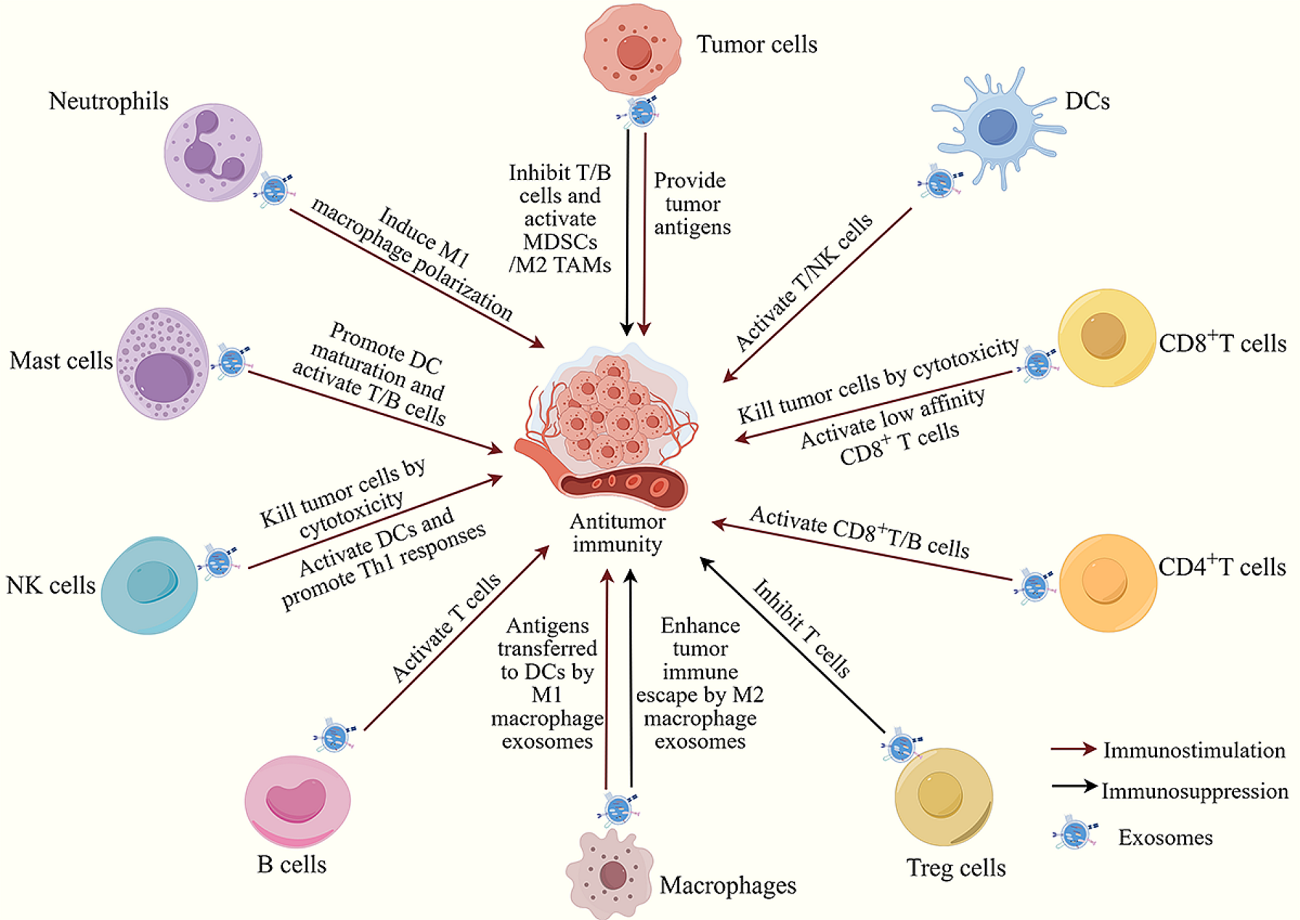
Immunomodulatory capability of tumor cell and immune cell-derived exosomes (By Figdraw)

Exosomes derived from tumor and immune cells have different essential functions in terms of shaping the cancer immune cycle, including antigen processing and presentation, antigen transfer, innate and adaptive immune activation, and immune suppression [101] (Fig. 3). Clarifying the intrinsic immunomodulatory capacity of exosomes derived from different cells and using exosomes as carriers to initiate anticancer immune responses or deliver anticancer drugs could help to further improve drug efficacy.

Tumor-derived exosomes (TEXs) carry specific tumor antigens, antigen-presenting molecules, and co-stimulatory molecules that activate anti-tumor immune responses. For example, TEXs carrying HSP70 induce NK cell-specific migration and cytolytic activity [102]. Meanwhile, TEXs expressing Rab27 enhance the expression of MHC II and the co-stimulatory molecules CD80 and CD86 in DCs, inducing DC maturation and promoting T cell proliferation [103]. However, TEXs encapsulate different contents, contributing to the complexity of their functions. TEXs can disrupt host immunity by inhibiting antigen recognition and presentation by DCs [104], promoting MDSC activation and differentiation [105], inducing the polarization of TAMs toward an immunosuppressive phenotype [106], and interfering with T cell proliferation and activation [107, 108]. Furthermore, in addition to promoting tumor immune escape, TEXs are also involved in various aspects of tumor progression by communicating with adjacent or distant cells, including the establishment of pre-metastatic niches, anti-apoptosis, angiogenesis, promotion of inflammatory response, tumor growth, and drug resistance [109, 110]. Consequently, the safety of utilizing TEXs as carriers warrants further examination.

Exosomes derived from immune cells serve as crucial mediators of both innate and adaptive immune responses. DC-derived exosomes (DEXs), enriched in MHC I, MHC II, CD80, CD86, ICAM-1, HSP70, and HSP90, play a key role in antigen presentation, thereby triggering immune responses based on both CD4^+^ and CD8^+^ T cells [111]. Additionally, DEXs transport functional peptide–MHC complexes to adjacent unresponsive DCs, enhancing the proliferation of DCs with specific peptides to facilitate widespread T cell activation and amplify immune responses [112]. Furthermore, DEXs express IL-15Rα, NKG2D ligand, and the TNF superfamily ligand and can directly bind to NK cell surface receptors and induce NK cell activation [113, 114]. T cell-derived exosomes have different roles in immunomodulation depending on their phenotype. For example, exosomes secreted by antigenically fully activated CD8^+^ T cells induce the activation of low-affinity CD8^+^ T cells, which are involved in tumor cell killing [115]. Moreover, CD4^+^ T cell-derived exosomes mediate anti-tumor cellular immunity, by activating CD8^+^ T cells, and enhance humoral immunity by promoting B cell proliferation, activation, and antibody production [116, 117]. In contrast, Treg exosomes express CD73 [118], carry specific miRNAs and iNOS [119], and synergize with cytokines to suppress the immune response [120]. Another study showed that exosomes from M1-like macrophages partially deliver phagocytosed antigens to DCs via a ceramide-dependent pathway, thus enhancing T cell responses [121]. NK cell exosomes carrying several specific miRNAs (miR-10b-5p, miR-92a-3p, and miR-155-5p) target molecules involved in the Th1 immune response and increase antigen presentation and co-stimulation through monocyte and monocyte-derived DC activation [122]. In addition, the roles of exosomes from B cells, mast cells, and neutrophils in the tumor immune response have been established [11, 123].

## Preparation of exosomes as drug delivery systems

### Isolation and purification

Given the characteristics of exosomes, such as their mass density, particle size, and surface protein markers, various strategies are available to isolate them from biofluids or cell culture supernatants. These include ultracentrifugation (UC), density gradient centrifugation (DG), ultrafiltration (UF), size exclusion chromatography (SEC), immunoaffinity capture, polymer precipitation, and microfluidics (Table 1).

UC is the most commonly used exosome isolation method and is the “gold standard” [124]. It involves two steps, as follows: (1) low-to-medium-speed centrifugation to remove cells, cellular debris, and large vesicles, and (2) ultra-high-speed centrifugation at 100,000 × g to obtain exosomes. UC is simple, technologically established, and suitable for processing large-dose samples. However, exosomes can be damaged by the high centrifugal forces, and impurities of a similar size and density might be co-separated with them [125–127]. Consequently, to enhance exosome purity, researchers developed DG through the introduction of separation media, such as sucrose and iodixanol. However, this method is cumbersome and time-consuming, limiting its efficiency [128]. Based on a difference in particle size, exosomes can be separated via UF or SEC. UF selectively separates samples through ultrafiltration membranes with different molecular-weight cutoffs. Moreover, it is a simple and low-cost method, but the membranes are susceptible to clogging, which causes exosome loss, and shear forces can damage the exosomes [129]. The principle of SEC is that large molecules cannot enter the gel pores and are eluted along the porous gel voids, while small molecules enter the gel pores and are eluted for a longer time. SEC is based on the principle of gravity flow, and the resulting exosomes retain a high degree of structural integrity and biological activity, with better recovery and purity than those with UC [130, 131]. However, the co-elution of similar-sized lipoproteins and the need for additional exosome enrichment pose challenges for the large-scale commercial application of SEC [132]. Immunoaffinity capture can be used to isolate exosomes based on interactions between their marker proteins (e.g., CD9, CD63, CD81, and ALIX, etc.) and specific antibodies. In this method, only exosomes that express proteins recognized by the antibody can be captured, and thus, the yield is low and isolation depends on the quality and specificity of the antibody. However, compared to other methods, immunoaffinity capture yields exosomes with a higher purity and can be used to isolate specific subtypes of exosomes [133, 134]. In addition, the binding of polyethylene glycol to water molecules and the formation of a hydrophobic microenvironment reduces exosome solubility and allows them to precipitate [135]. This polymer precipitation method is simple, but the exosomes obtained have more impurities and the polymer is difficult to remove [136]. Finally, microfluidics comprise a micro-sized horizontal separation technique based on the physicochemical characteristics of exosomes, and this has advantages over conventional methods, including its automated operation, precise control of flow conditions, high purity, and short time. However, microfluidics approaches require expensive and sophisticated equipment and are not suitable for large-scale applications [137, 138]. In conclusion, we should flexibly choose one or more methods for exosome isolation depending on the sample types, sources, volumes, and downstream applications, in addition to considering the yield, purity, cost, and time.

**Table 1:** Comparison of exosome isolation and purification methods

Methods	Principle	Time	Purity	Yield	Advantages	Limitations
UC	Mass density	Long	Moderate	Moderate	• Gold standard • Simple • Suitable for large-dose samples	• Expensive equipment • Time-consuming • Exosome damage and contamination
DG	Mass density	Long	High	Low	• High purity	• Low throughput • Complex operation • Time-consuming
UF	Size	Short	Moderate	Moderate	• Simple • Low cost	• Membrane clogging • Exosome damage and contamination
SEC	Size	Short	High	High	• High purity • Maintaining exosome activity and structure	• Potential contamination • Extra exosome enrichment methods
Immunoaffinity capture	Markers binding to antibodies	Short	High	Low	• High purity • Suitable for separating source-specific exosomes	• High cost • Extra elution steps • Exosome markers must be optimized
Polymer precipitation	Polymer binding to water molecules	Long	Low	High	• Simple and convenient • High yield	• Exosome contamination • Extra exosome purification steps
Microfluidics	Size, density, immunoaffinity	Short	High	Low	• High efficiency • Easily automated	• Advanced equipment • Low sample capacity

### Drug loading

The process of loading drugs into exosomes is crucial for the development of exosome-based drug delivery systems. Maintaining drug activity and effective loading without compromising exosome integrity is the primary concern when selecting the optimal loading method. Drug loading strategies for exosomes can be primarily divided into endogenous and exogenous. Subsequently, the characteristics of these different loading methods will be discussed.

Endogenous loading refers to therapeutic molecules being imported into the source cells via transfection, co-incubation, or electroporation and then loaded into exosomes through a cell sorting mechanism. Endogenous loading has a wide range of applications and does not destroy exosomes, but the process is complex and time-consuming [139, 140]. Crucially, with endogenous loading, control over exosome yields and therapeutic molecule encapsulation is lacking, generally resulting in low loading efficiency [141]. Recently, Yang et al. [142] introduced a cellular-nanoporation technique for the large-scale production of exosomes encapsulating therapeutic mRNAs and targeted peptides. Compared to results with electroporation and other exosome production strategies, this method can be used to produce up to 50-fold more exosomes, increase mRNA transcripts by more than 10^3^-fold, and result in better therapeutic efficacy based on glioma models.

In exogenous loading, the exosome is first isolated, and then, the therapeutic molecule is directly loaded onto the exosome membrane or into the lumen. This method offers controllable encapsulation efficiency and loading capacity, generally outperforming endogenous loading [143]. Various loading methods have been developed, such as incubation, freeze–thaw, sonication, extrusion, electroporation, and permeabilization. Incubation is used to passively load drugs via concentration gradients without compromising the integrity of the exosome membrane. However, this still mainly applies to hydrophobic small molecules, and the loading efficiency is affected by the drug concentration, pH, and temperature, among other factors [144, 145]. For freeze–thaw, rapid temperature changes are employed for active drug loading into exosomes; it is simple but has a low loading rate and can lead to exosome aggregation [143, 146]. In contrast, sonication and extrusion result in higher loading efficiencies [147, 148], but they can damage the exosome membrane and reduce drug activity [149]. Similarly, electroporation causes the formation of several temporary hydrophilic pores on the exosome membrane using an applied electric field, permitting the entry of drugs into the exosome. Studies have shown that the loading efficiency of electroporation is three times that of incubation but much less than that of sonication and that it causes exosome damage and aggregation [145, 148, 150]. Surfactants, such as saponins, enhance exosome membrane permeability for drug loading, which is known as the permeabilization method. However, surfactants can alter drug activity and are associated with a risk of hemolysis and cytotoxicity, necessitating further purification [144, 146, 151]. In addition, dialysis [152] and in situ synthesis [153] are also employed for drug loading, albeit less commonly than other methods. Table 2 outlines the principles, advantages, disadvantages, and applicable types of therapeutic molecules of commonly used drug loading methods.

**Table 2:** Comparison of exosome drug loading methods

Type	Methods	Principle	Advantages	Limitations	Drug type
Endogenous loading	Co-incubation	Passive diffusion into cell membranes	• Simple • Exosome integrity	• Low efficiency • Parent cell toxicity • For lipophilic molecules only	• Drugs • Nanoparticle
	Electroporation	High-voltage pulses produce diffusion orifices	• Exosome integrity	• Low efficiency • Need process optimization	• Drugs • Nanoparticle
	Transfection	Gene editing	• Desired molecule overexpression • Exosome integrity	• Low efficiency • Transfectant toxicity • Technically complex	• Nucleic acids • Proteins or peptides
Exogenous loading	Incubation	Passive diffusion into exosome membranes	• Simple • Exosome integrity	• Low efficiency • For lipophilic molecules only	• Drugs • Nanoparticle • Nucleic acids • Proteins or peptides
	Freeze-thaw	Membrane fusion	• Simple	• Low efficiency • Exosome aggregation	• Proteins or peptides
	Sonication	Mechanical shears produce diffusion orifices	• High efficiency	• Exosome aggregation • Exosome damage	• Drugs • Nanoparticle • Proteins or peptides
	Extrusion	Physically generate diffusion orifices	• High efficiency	• Exosome aggregation • Exosome damage	• Drugs • Proteins or peptides
	Electroporation	High-voltage pulses produce diffusion orifices	• Fast and easy	• Exosome aggregation • Exosome damage	• Drugs • Nanoparticle • Nucleic acids • Proteins or peptides
	Permeabilization	Surfactants generate diffusion orifices	• Simple • High efficiency	• Hemolysis • Toxicity • Drug activity damage • Purification required	• Nanoparticle • Proteins or peptides
	Dialysis	Concentration or PH gradients	• Simple • Improved efficiency	• Exosome aggregation • Protein degradation	• Drugs • Nucleic acids
	In-situ synthesis	Chemical reaction	• Exosome integrity	• Complicated process	• Nanoparticle

## Exosome-based immunotherapy strategies for gliomas

**Fig. 4:**
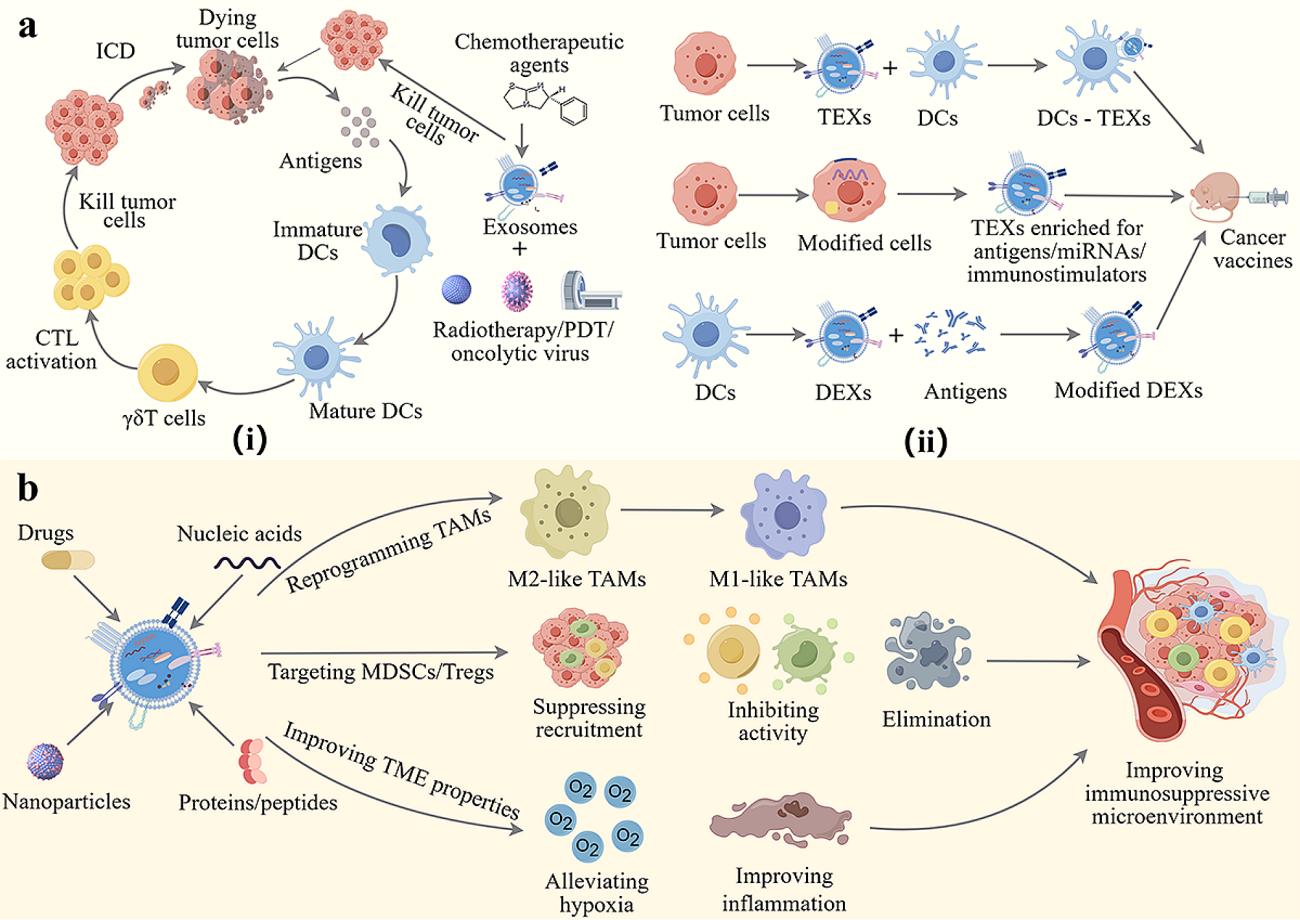
Exosome-based immunotherapy strategies for gliomas (By Figdraw). **a** Improvement of tumor low immunogenicity. (i) Exosomes precisely deliver chemotherapeutic drugs to promote antigen release and trigger immunogenic cell death; (ii) Exosomes from tumor and immune cells as cancer vaccines to enhance antigen expression and presentation. **b** Improvement of the immunosuppressive microenvironment, including reprogramming TAMs, targeting MDSCs/Tregs, and improving TME physicochemical properties

To address the challenges associated with glioma immunotherapy, we propose strategies using exosome delivery systems to boost immune efficacy by enhancing immunogenicity and reversing the immunosuppressive microenvironment (Fig. 4). Additionally, we provide a summary of the types and effectiveness of current exosome-based drug delivery systems for glioma immunotherapy (Table 3).

### Cascade modulation to improve low tumor immunogenicity

#### Targeted delivery of chemotherapeutic agents to promote antigen release

Tumor cell death resulting in the release of sufficient antigens is the basis for triggering specific immune responses. Chemotherapy can kill tumor cells, produce large amounts of antigens, and induce immunogenic cell death (ICD). However, the inefficient targeted delivery of chemotherapeutic agents impairs their cytotoxic effects on tumors, leading to reduced antigen release and limiting the effects of ICD, which can even cause other severe side effects in organs. Therefore, exosome-based drug delivery systems could be a promising strategy for enhancing the efficacy of chemotherapeutic agents.

Transmembrane proteins on the surfaces of exosomes (such as integrins, tetraspanins, and ICAM-1) confer intrinsic cell- or tissue-targeting properties to exosomes [101]. For example, exosome-encapsulated doxorubicin (DOX) shows faster cellular uptake and accumulates at higher concentrations than free DOX and liposomal DOX in multiple cell lines [154]. Moreover, brain endothelial cell-derived exosomes carrying DOX nanoparticles can be used to deliver drugs to the tumor region and cause their accumulation, inducing tumor cell apoptosis and ICD, leading to DC maturation and CTL infiltration, and enhancing immune responses in GBM mouse models [155]. Based on the intrinsic inflammatory chemotactic capacity of neutrophils, Wang et al. [156] constructed a neutrophil-exosome system (NEs-Exos) to deliver DOX. Substantial evidence suggested that NEs-Exos can cross the BBB and respond chemotactically to inflammatory stimuli, targeting the inflammatory sites of gliomas. Genetic engineering or chemical modification to introduce tissue- or cell-specific ligands onto the exosome surface can further enhance targeting. Angiopep-2 (ANG) has a high affinity for low-density lipoprotein receptor-related protein-1, which is highly expressed in glioma and BBB endothelial cells. Exosomes co-modified using ANG and trans-activator of transcription peptides, highly potent cell-penetrating peptides, can significantly increase DOX penetration into the tumor region through dual targeting for optimal glioma therapy [157]. Moreover, c(RGDyk) modifications on the surfaces of embryonic stem cell-derived exosomes and simultaneous paclitaxel loading were used to treat GBM; here, the modified exosomes significantly improved the therapeutic efficacy of paclitaxel through enhanced targeting compared to that with unmodified exosomes and free paclitaxel [158]. Other tumor-targeting molecules, such as neuropilin-1-targeting peptide [159], heme oxygenase-1 specific short peptide [160], and transferrin receptor-binding peptide [161], have also been experimentally used for exosome surface modification to enhance drug delivery. Thus, exosomes, based on intrinsic targeting or obtained through engineered modifications contribute to chemotherapeutic agent delivery, enhance tumor cell killing, and ultimately facilitate the release of many tumor antigens to enhance immune efficacy.

Mono-chemotherapy often results in resistance and causes insufficient antigen release [162]. Therapies such as radiotherapy, photodynamic therapy, photodynamic therapy (PDT), and oncolytic viruses can also cause apoptosis in tumor cells, resulting in the release of antigens and triggering ICD [163, 164]. Therefore, combination therapy might be a practical anti-tumor approach. Wang et al. [165] fabricated Rg3-modified homologous engineered exosomes for co-delivery of the chemotherapeutic agent arsenic trioxide (ATO) and the photosensitizer chlorin e6 (Ce6). Specifically, it was determined that ATO and Ce6 could be delivered to the tumor area. Under laser irradiation, Ce6-triggered PDT destroys tumor cells and results in the release of more tumor antigens, synergistically improving ATO efficacy. Similarly, a recent study reported a novel strategy for multilevel cascade GBM therapy with M1 macrophage-derived extracellular vesicles (M1EVs) co-loaded with the chemo excitation source CPPO, Ce6, and hypoxia-activated prodrug AQ4N. In this system, M1EVs were found to modulate the TME to increase the level of hydrogen peroxide, which reacts with CPPO to generate chemical energy to activate Ce6 and produce reactive oxygen species for PDT. At the same time, this reaction consumes oxygen to activate the AQ4N toxicity [166]. In addition, in lung cancer-model mice, exosome-mediated combination therapy with oncolytic viruses and paclitaxel significantly induced ICD and T cell infiltration [167], and its application to gliomas might also have significant potential.

#### Increased antigen expression and presentation with cancer vaccines

TEXs can be used as antigenic vehicles for cancer immunotherapy, for example as cancer vaccines, to elicit immune responses. Harshyne et al. [168] injected different doses of GM-Exos into mice to establish an immune model. They found that high doses of GM-Exos induced protective immunity in mice transplanted with GL261 cells, causing less tumor growth in vivo. This protective effect was associated with enhanced T cell activity and specific antibody production induced by the GM-Exos. However, the immune response elicited by TEXs is relatively weak, requiring the preparation of vaccines with higher immunogenicity. Vaccines comprising DCs loaded with tumor-associated antigens or tumor cell lysates are being widely developed. TEXs have many advantages over traditional antigens, and these include the enrichment of tumor antigens, a strong antigen-presenting phenotype, and easy uptake by DCs and guidance to the antigen-processing region; therefore, these could serve as antigenic vectors for DC vaccines [169]. One study demonstrated that DCs co-incubated with TEXs stimulate the differentiation of T lymphocytes into antigen-specific CTLs, with better glioma cell-killing ability than DCs loaded with cell lysates [170]. Based on these findings, Liu et al. [171] constructed a novel DC vaccine using TEXs in combination with an invariant natural killer T cell adjuvant, which could modulate the release of immunosuppressive/immunostimulatory factors, deregulate the precancer immunosuppressive microenvironment, and induce a solid antigen-specific CTL response against GBM cells.

In addition, the artificial modification of TEXs to enrich tumor antigens, immunostimulatory molecules, and miRNAs enables the fabrication of cell-free cancer vaccines with good immunogenicity and immunostimulatory capacity [169]. To date, engineered TEX vaccines for gliomas have been less studied, but several strategies used to modify TEXs have been developed and have shown good efficacy for other cancers [172]. For example, leukemia cells were transfected with lentiviral vectors encoding B7 co-stimulatory molecules, and the exosomes (LEX-8086) obtained expressed high levels of CD80 and CD86. LEX-8086 was effective in inducing CD4^+^ T cell proliferation, Th1 cytokine secretion, and a CTL response [173]. Moreover, heat-treated tumor cells can produce exosomes that are highly enriched in HSP70 (HS-TEXs). HS-TEXs, an MHC-independent vaccine, increase IgG2a and IFN-γ production, triggering a strong Th1-type immune response [174]. INF-γ-modified exosome vaccines (INF-γ-TEXs), obtained via protein-anchoring, increase the number of M1-like macrophages and the proportion of IFN-γ^+^ CD8^+^ T cells and decrease the proportion of Tregs [175]. In conclusion, these studies suggest that fully deciphering the immunostimulatory/immunosuppressive mechanisms of TEXs and modifying them appropriately could be a valuable immunotherapeutic approach for gliomas.

DEXs express more MHC antigens than DCs and are easy to modify genetically, have a long half-life, and can be used as a biological nano-vaccine to activate the immune response, instead of DCs [111, 176]. Bu et al. [177] found that DEXs containing chaperonin-rich cell lysates induce a strong anti-tumor immune response in mice with glioma by maintaining T cell activation. Moreover, DEXs loaded with neoantigens significantly inhibit tumor growth, preventively delay tumor occurrence, and establish long-term immune memory-mediated protection by inducing broad-spectrum T and B cell immune responses [178]. Recently, Zuo et al. co-loaded hepatocellular carcinoma-targeting peptides, α-fetoprotein epitopes, and an immune adjuvant on the surface of DEXs to prepare a novel bio-nano-vaccine with universal applicability for DC recruitment and activation. The vaccine was found to promote the recruitment and activation of endogenous DCs, as well as tumor neoantigen cross-presentation, to induce T cell responses and activate the innate immune response, providing an innovative approach for developing personalized cancer vaccines [179]. In addition, M1-like macrophage exosomes can be taken up by local DCs and macrophages and cause the release of Th1 cytokines, inducing more robust CTL responses and synergistically enhancing the efficacy of nanoparticle vaccines, suggesting that they have the potential to serve as adjuvants for cancer vaccines [180].

Although the applications of exosome vaccines in glioma are still limited, there is a relatively solid theoretical foundation and reliable methods to prepare exosome vaccines for glioma based on their widespread use in other cancers. In the future, we should continue to explore new approaches to increase exosome immunogenicity and immune activation capacity and overcome host immunosuppression to obtain effective exosome vaccines for glioma.

### Multiple targets to synergistically improve the immunosuppressive microenvironment

#### Reprogramming TAMs

TAMs have two phenotypes, specifically M1-like TAMs obtained via INF-γ and Toll-like receptor 4 stimulation and M2-like macrophages derived from IL-4, IL-10, and IL-13 stimulation [181]. M1-like TAMs secrete TNF-α, IL-6, and IL-12 to promote anti-tumor immune responses. However, the M2 phenotype is predominant among TAMs and is associated with local and systemic immunosuppression in glioma patients [182]. Therefore, reprogramming M2 tumor-promoting phenotypes into M1 anti-tumor phenotypes is a promising strategy for cancer therapy. However, owing to potential side effects, most approaches do not allow for the in situ reprogramming of TAMs. Based on the advantages of exosomes as transporter vehicles, attempts have been made to precisely deliver various therapeutic agents to reprogram TAMs and reduce immunosuppression.

Li et al. [183] constructed a dual delivery system for GBM immunotherapy and evaluated its effect on tumor-bearing mice. First, they prepared T7 peptide-modified exosomes (T7-Exos) and loaded Galectin‑9 siRNA (siGalectin-9) into the exosomes via electroporation. The results showed that T7-Exos could effectively deliver siGalectin-9 to GBM cells. Moreover, siGalectin-9 was dependent on activation of the TLR7–IRF5 pathway, which promoted macrophage polarization to the M1 phenotype, increased macrophage phagocytosis of glioma cells, and activated the immune response. Signal transducer and activator of transcription 3 (STAT3) is a crucial transcription factor that induces macrophage polarization towards the M2 phenotype [181]. Cui et al. [184] reported the self-assembly of tanshinone IIA (TanIIA) and glycyrrhizic acid (GL), which are the inhibitors of STAT3, to form TanIIA-GL nanogel micelles (TGM). Serum-derived exosomes encapsulated with TGM and surface-modified with the immunoadjuvant CpG oligonucleotides were further used to construct a bionic nanodelivery system (CpG-EXO/TGM). CpG-EXO/TGM could be effectively taken up by glioma cells, promoting TAM reprogramming, DC maturation, and CTL activation and activating immune memory cells to prevent tumor recurrence in glioma recurrence models. Similarly, the encapsulation of CpG-STAT3 antisense oligonucleotide using neural stem cell-derived exosomes was found to stimulate macrophage immunoactivity, induce NF-κB signaling and IL-12 production, and inhibit glioma growth [185]. High-molecular weight hyaluronic acid (HMW-HA) inhibits M1-like macrophage polarization and enhances M2-like macrophage polarization [186]. Human hyaluronidase delivered by folic acid-modified exosomes can enhance antitumor efficacy by degrading HMW-HA to promote the polarization of TAMs to the M1 phenotype [187]. In addition, ginseng-derived exosome-like nanoparticles (GENs) carrying multiple chemical cargoes can penetrate the BBB and target glioma cells, recruit M1-like macrophages, and downregulate the M2-like macrophage phenotype, suggesting that GENs are promising candidates for the development of nanocarriers for glioma immunotherapy [188].

Further, M1-like macrophages can secrete the necessary molecules to consistently drive an M2-to-M1 phenotypic conversion, suggesting the feasibility of exploiting M1-like macrophage-derived exosomes to mediate TAM reprogramming [189]. Researchers found that exosome-mimetic nanovesicles from M1-like macrophages can induce the repolarization of M2-like TAMs to the M1 phenotype by modulating the expression profile of miRNAs and mRNAs, which in turn results in the release of pro-inflammatory cytokines to induce anti-tumor immune responses and synergistically enhance the efficacy of ICIs in glioma models [190]. Compared to those on M1-like TAMs, IL4 receptor (IL4R) is expressed at higher levels on M2-like TAMs and is involved in M2 macrophage polarization in response to the cytokine IL-4 [191]. M1 exosomes were transfected with NF-κB p50 siRNA and miR-511-3p to enhance M1 polarization and were surface-modified using IL-4R-binding peptides to target the IL4 receptor (IL4R-Exos(si/mi)). IL4R-Exos(si/mi) can be efficiently internalized by M2-like TAMs and reprogrammed to M1-like macrophages to enhance anti-tumor immunity [192]. In conclusion, exosome-based drug delivery systems can reverse the immunosuppressive microenvironment by reprogramming TAMs into anti-tumor phenotypes to achieve efficient therapy for gliomas.

#### Targeting MDSCs and Tregs

Based on the immunosuppressive role of MDSCs and Tregs in the TME, researchers have focused on developing immunotherapies targeting these cells to restore the anti-tumor immune response. This includes (i) blocking the recruitment of MDSCs/Tregs, (ii) deregulating the immunosuppressive activity of MDSCs/Tregs, and (iii) depleting MDSCs/Tregs. Here, we present the current strategies used to target MDSCs/Tregs in glioma therapy, to load these therapeutic agents into exosomes and achieve precision therapy.

##### Targeting MDSCs

The CCL2–CCR2 axis is an essential target for blocking the migration of MDSCs to tumors. CCX872, an antagonist of CCR2, reduces MDSC infiltration and enhances the efficiency of ICIs in glioma-bearing mice [193]. Moreover, a macrophage migration inhibitory factor inhibitor targets a monocytic subset of MDSCs via the CD47 receptor, suppressing downstream MCP-1 signaling and inhibiting MDSC recruitment and expansion [194]. In addition, other chemokines that block MDSC recruitment, such as CCR5 antagonists [195], VEGF antagonists [196], and CXCR2 antagonists [197], are also being actively studied. Developing exosomes containing these blockers for glioma therapy might result in excellent efficacy.

Promoting the differentiation of MDSCs to mature myeloid cells is the most direct and effective way to reverse their immunosuppressive activity. Vitamin A and its metabolites, vitamin D, STAT3 inhibitors, and TLR agonists can mediate the differentiation of MDSCs [44]. All-trans retinoic acid is a vitamin A derivative that can effectively inhibit glioma growth when applied alone [198] or in combination with a DNA demethylating agent [199] and interferon-gamma [200]. Aberrant activation of the cyclooxygenase 2 (COX2)/prostaglandin E2 (PGE2)/PGE2 receptor axis is vital for maintaining the immunosuppressive activity of MDSCs. Researchers found that the COX2 inhibitors acetylsalicylic acid or celecoxib inhibit MDSC activation by decreasing PGE2 production and retard the growth of glioma [201]. Currently, combination therapeutic strategies are under investigation, and exosome-based specific delivery systems could offer a promising means to inhibit MDSCs.

In addition, MDSCs can be exhausted by specific antibodies targeting MDSCs and chemotherapeutic agents. An anti-CD33 antibody, as well as an agonistic DR5 antibody, resulted in the favorable induction of apoptosis in MDSCs [202]. Moreover, some low-dose chemotherapeutic agents (5-fluorouracil and gemcitabine, etc.) can directly exert cytotoxic effects, mediating the depletion of MDSCs. A phase 0/I clinical trial found that the combination of capecitabine, a prodrug of 5-fluorouracil, and bevacizumab reduces circulating MDSC levels in GBM patients [203].

However, when using these therapeutic strategies, problems such as high toxicity, low permeability, and delivery efficiency could be generally encountered. Exosomes, as a powerful drug-loading system, are expected to achieve better therapeutic effects. In fact, researchers have already made some attempts to implement this strategy. Qiu et al. found that modified DEXs loaded with miR-21 inhibitors synergistically improve the therapeutic efficacy of ICIs and prolong the survival of glioma-bearing mice. This was associated with its ability to target tumor mesenchymal stem cells and disrupt the miR-21/SP1/DNMT1 positive feedback loop and reduce MDSC activation by decreasing CD73 expression on MDSCs [204]. Furthermore, glioma-derived extracellular vesicles overexpressing basic leucine zipper ATF-like transcription factor 2 inhibit the recruitment of MDSCs to limit glioma growth [205]. Therefore, based on the aforementioned studies, developing exosome delivery systems targeting MDSCs could be a promising approach.

##### Targeting Tregs

Similarly, treatment strategies based on Tregs have been extensively studied. Targeting the glucocorticoid-induced TNFR-related receptor (GITR) in Tregs using αGITR promotes Treg differentiation into effector T cells and attenuates treatment resistance in GBM models [206]. Loading an anti-GITR antibody and catalase into nanoparticles consisting of a photothermal agent and photosensitizer induces a 4.3-fold reduction in Tregs and exerts a strong antitumor effect [207]. Moreover, heat stress-treated TEXs stimulate DCs to secrete IL-6 to block the TGF-β1-induced differentiation of Tregs and promote Treg conversion to Th17 cells [208]. A detailed summary of cancer therapeutic strategies targeting Tregs has been presented [209], which is an important guide for the development of exosome-based glioma nano-immunotherapies.

#### Improving TME physicochemical properties

Hypoxia is a crucial trigger of glioma invasion, anti-apoptotic processes, angiogenesis, and resistance to radiotherapy and chemotherapy [210]. Hypoxic glioma niches attract and sequester TAMs and CTLs and reprogram them toward an immunosuppressed state [211]. Liu et al. [212] developed a glioma-targeted transport system to enhance sonodynamic therapy by alleviating tumor hypoxia. They first encapsulated catalase into silica nanoparticles (CAT@SiO_2_) and then loaded the sonosensitizer indocyanine green to prepare a biodegradable nano platform (CSIs). The CSIs were further encapsulated with AS1411 aptamer-modified macrophage exosomes to form a delivery system (termed CSI@ Ex-A) with highly efficient BBB-infiltration and tumor-targeting capabilities. At the tumor site, highly expressed glutathione triggered the biodegradation of CSIs, and the catalase that was released catalyzed the production of O_2_ from H_2_O_2_ in the tumor, thus alleviating the hypoxic microenvironment and improving the efficiency of sonodynamic therapy. In addition, CSI@Ex-A effectively inhibited glioma metastasis in vivo, which could be related to the alleviation of tumor hypoxia and the suppression of the activation of hypoxia-inducible factor 1α. Moreover, chronic persistent inflammation confers immune-escape ability in glioma, inducing immune tolerance to numerous therapies. Therefore, glioma therapy can be achieved by suppressing the inflammatory state in the TME. Extracellular vesicles overexpressing esophageal cancer related gene-4 significantly inhibit the expression of inflammatory cytokines (IL-1β, IL-6, IL-8) and activate the p38–AMPK signaling pathway to suppress the inflammatory response and induce anti-tumor effects in vitro and in vivo [213]. Exosomes loaded with STAT3 inhibitors that enter the brain by crossing the BBB, after nasal administration, are selectively taken up by microglia and inhibit the expression of inflammatory cytokines, such as IL-1β and IL-6, ultimately retarding glioma cell growth [214]. In summary, improving hypoxia and chronic inflammation in the TME could also be a strategy to reverse immunosuppression and enhance therapeutic efficacy.

**Table 3:** Exosome-based drug delivery systems for glioma immunotherapy

Delivery systems	Exosome Source	Valid Cargo	Therapy strategy	Model type	Therapeutic effect	Immune effect	Ref
Exo@ DOX	bEnd.3 cells	DOX	ICD	GL261 cells and GL261 tumor mice	• Increased tumor cell apoptosis • Prolonged mice survival	• DC maturation • CTL activation • Cytokine changes	155
Rg3-Exo/ATO@Ce6	GL261 cells	ATO, Rg3, and Ce6	ICD and reprogram TAMs	GL261 cells and GL261 tumor mice	• Induced tumor cell apoptosis • Inhibited tumor growth • Prolonged mice survival	• DC maturation • T cell activation • TAM polarization • Cytokine changes	165
CCA-M1EVs	M1 macrophage	CPPO, Ce6, and AQ4N	ICD and reprogram TAMs	U87 tumor spheroids, U87MG xenografts, and GBM PDX mice	• Inhibited tumor growth • Prolonged mice survival	• Modulated M2-to-M1 polarization • Cytokine changes	166
DEXs (CRCL-GL261)	DCs	CRCL	Cancer vaccine	GL261 cells and GL261 tumor mice	• Inhibited tumor growth • Prolonged mice survival	• T cell activation and proliferation • Cytokine changes	177
T7-Exo/ Galectin‑ 9 siRNA	HEK293T cells	Galectin‑ 9 siRNA	Reprogram TAMs	GL261 cells and GL261 tumor mice	• Inhibited tumor growth	• Polarized macrophages to M1 phenotype	183
CpG-EXO/TGM	Serum	TanII, GL, and CpG	Reprogram TAMs	U87 and GL261 tumor spheroids and GL261 tumor mice	• Increased tumor cell apoptosis • Prolonged mice survival • Prevented recurrence	• TAM polarization • DC maturation • CTL activation • Cytokine changes	184
ANG -modified DEXs loaded with miR21 inhibitor	DCs	miR21 inhibitor	Inhibit MDSC activity	GL261 tumor mice	• Prolonged mice survival	• Reduced MDSC percentage and activity • Increased CD8 IFN-γ cells	204
CSI@Ex-A	Macrophage	CAT@ SiO_2_ and ICG	Alleviate hypoxia	U87 cells and U87 tumor mice	• Inhibited tumor growth and metastasis • Prolonged mice survival	• Generated oxygen • Inhibited HIF-1α activation	212
ECRG4-Exo	Human brain endothelial cells	ECRG4	Improve inflammation	U87MG and T98G cells and T98G tumor mice	• Inhibited tumor growth • Prolonged mice survival	• Inhibited inflammatory cytokine levels	213
Exo-JSI124	Mouse lymphoma cells	STAT3 inhibitor (JSI124)	Improve inflammation	GL261 tumor mice	• Inhibited tumor growth • Prolonged mice survival	• Induced microglia apoptosis • Inhibited inflammatory cytokine expression	214

## Clinical translation and challenge

According to Clinicaltrials.gov (https://clinicaltrials.gov/) [215], there are four clinical trials based on exosomes as drug delivery systems for cancer treatment (Table 4), all of which are at an early stage. Although exosomes for drug delivery have been extensively studied, they still face challenges in clinical translation. First, there is an urgent need to develop standardized and industrialized methods for exosome production, isolation, and purification for the large-scale preparation of clinical-grade exosome products. Exosomes are heterogeneous, and small changes in the production process can affect their yield and activity. However, a gold standard for exosome preparation is lacking, and relatively low yields limit the further clinical applications of exosomes. Researchers have already enhanced exosome secretion in preclinical studies using physical stimulation, molecular interference, environmental factors, and external inducers [216], but extending these findings to the clinic still requires the consideration of various aspects, such as safety, bioactivity, efficiency, and stability. In addition, the exosomes collected using existing separation and purification techniques do not meet the standards for clinical use. Second, the safety of exosomes should be considered. Exosomes of different cell origins have different functions in cancer immunomodulation, and the mechanism underlying the effects of exosomes on cancer immunity should be thoroughly clarified. Especially, TEXs, despite their homing properties and immunogenicity, also contribute to tumor progression through epithelial–mesenchymal transition, angiogenesis, and immune escape. Mesenchymal stem cells can be used for safe and stable exosome production [217]. However, these exosomes lack antigens and antigen-presenting molecules, when compared to exosomes derived from immune and tumor cells, which limits their use in cancer vaccines. In addition, serious safety issues, such as cytokine release syndrome, could arise from the use of exosome drugs [218]. Third, there are still some limitations to the engineering and modification of exosomes. Genetic engineering or chemical modifications can further enhance exosome-specific targeting capabilities. However, genetic engineering is limited to targeting motifs that can be genetically encoded. Moreover, chemical modifications often affect the functions of exosomes, and the complexity of the exosome structure reduces the efficiency of the reaction [219]. In addition, there is no standardized drug-loading technique. Several commonly used drug-loading strategies have different disadvantages and cannot simultaneously combine the properties of high loading efficiency, limited exosome damage, and easy operation. Finally, exosome storage is also a pressing issue. Different storage conditions will affect the exosome morphological structure, protein content, and biological activity. Currently, it is believed that exosomes are more stable at − 80 °C and can maintain clinical availability after 5 months [220, 221]. However, high storage and transportation costs can restrict generalization. In summary, we still need to improve on existing technologies or develop new ones to overcome the problems associated with exosome-based drug delivery systems in terms of their clinical translation, large-scale production, stable preparation and storage, and quality control.

**Table 4:** Clinical trials of exosomes as drug delivery systems

Item number	Therapy strategy	Cancer type	Case	Status	Phase	Start time
NCT01159288	Tumor antigen-loaded dendritic cell-derived exosomes	Non-small cell lung cancer	41	Completed	Phase 2	May 2010
NCT01294072	Plant-derived exosomes loaded with curcumin	Colon cancer	35	Recruiting	Not applicable	January 2011
NCT03608631	Mesenchymal stromal cell-derived exosomes loaded with KrasG12D siRNA	Pancreatic cancer	15	Active, not recruiting	Phase 1	January 2021
NCT05375604	Cell-derived exosomes loaded with STAT6 anti-sense oligonucleotide	Hepatocellular carcinoma and liver metastases from either primary gastric cancer or colorectal cancer	9	Terminated	Phase 1	May 2022

The data is obtained from https://clinicaltrials.gov/ [215]

## Conclusions

As a natural delivery system, exosomes have the unique advantages of low toxicity, targeting ability, and immunomodulatory properties, which can be exploited for cancer immunotherapy. Exosomes for the precise delivery of chemotherapeutic drugs, antigens, antigen-presenting molecules, and immunomodulatory molecules to tumors can overcome certain obstacles to existing glioma immunotherapy approaches, including the BBB, low immunogenicity, and an immunosuppressive microenvironment. Exosome-based immunotherapeutic strategies, including increasing antigen release, expression, and presentation, reprogramming TAMs, targeting Tregs and MDSCs, and improving the physicochemical properties of the TME, offer new hopes for effectively enhancing glioma treatment. Although the clinical translation of exosomes still faces many challenges, exosome-based drug delivery systems hold great promise for the glioma immunotherapy. With the continuous development and innovation of new technologies, exosome therapy will soon be at the forefront of glioma treatment.

## Data Availability

No datasets were generated or analysed during the current study.

## Abbreviations

ANG: Angiopep-2
APC: Antigen-presenting cell
APM: Antigen processing and presentation machinery
ARG1: Arginase
ATO: Arsenic trioxide
BBB: Blood–brain barrier
BMEC: Brain microvascular endothelial cell
Ce6: Chlorin e6
circRNAs: Circular RNAs
CNS: Central nervous system
COX2: Cyclooxygenase 2
CRCL: Chaperone-rich cell lysates
CTL: Cytotoxic T lymphocyte
DC: Dendritic cell
DEX: Dendritic cell-derived exosomes
DG: Density gradient centrifugation
DOX: Doxorubicin
e-: Expressed neoantigens
ECRG4: Esophageal cancer related gene-4
ESCRT: Endosomal sorting complex required for transport
GBM: Glioblastoma
GEN: Ginseng-derived exosome-like nanoparticle
GITR: Glucocorticoid-induced TNFR-related receptor
GL: Glycyrrhizic acid
GM-Exos: Glioma-derived exosomes
HIF-1α: Hypoxia-inducible factor 1α
HLA: Human leukocyte antigen
HMW-HA: High-molecular weight hyaluronic acid
HS-TEXs: Exosomes that are highly enriched in HSP70
ICAM-1: Intercellular adhesion molecule
ICD: Immunogenic cell death
ICG: Indocyanine green
ICI: Immune checkpoint inhibitor
INF-γ-TEXs: INF-γ-modified exosome vaccines
ILV: Intraluminal vesicle
IMC: Immature myeloid cell
iNOS: Inducible nitric oxide synthase
LEX8086: Exosomes enriched for B7 co-stimulatory molecules
lnRNAs: Long non-coding RNAs
M1EV: M1 macrophage-derived extracellular vesicle
MDSC: Myeloid-derived suppressor cell
MHC: Major histocompatibility complex
MVB: Multivesicular body
miRNAs: microRNAs
mRNAs: Messenger RNAs
NEs-Exos: Neutrophil–exosome system
NK: Natural killer
p-: Predicted neoantigens
PGE2: Prostaglandin E2
PDT: Photodynamic therapy
PDX: Patient-derived xenograft
SEC: Size exclusion chromatography
siGalectin-9: Galectin‑9 siRNA
STAT3: Signal transducer and activator of transcription 3
T7-Exos: T7 peptide-modified exosomes
TAM: Tumor-associated macrophage
TanIIA: Tanshinone IIA
TEX: Tumor-derived exosome
TGMs: TanIIA-GL nanogel micelles
TMB: Tumor mutational burden
TLR: Toll-like receptor
TME: Tumor microenvironment
Treg: Regulatory T cell
UC: Ultracentrifugation
UF: Ultrafiltration
